# Nobiletin Protects Endothelial Function in High-Fat Diet-Induced Obese Mice Through Activation of 5′ Adenosine Monophosphate-Activated Protein Kinase

**DOI:** 10.3390/nu18101564

**Published:** 2026-05-14

**Authors:** Qiaowen Deng, Yuehan Wang, Yifan Yang, Lingchao Miao, Chumin Zhong, Manqin Fu, Wai San Cheang

**Affiliations:** 1 State Key Laboratory of Mechanism and Quality of Chinese Medicine, University of Macau, Macau SAR, China; mc25499@um.edu.mo (Q.D.); yc17511@um.edu.mo (Y.W.); mc46239@um.edu.mo (Y.Y.); lingchaomiao@um.edu.mo (L.M.); 2 Institute of Chinese Medical Sciences, University of Macau, Macau SAR, China; 3 Jiangmen Palace Qiaobao Chenpi Health Industry Development, Inc., Jiangmen 529000, China; jmlgsp@foxmail.com; 4 Sericultural & Agri-Food Research Institute, Guangdong Academy of Agricultural Sciences/Key Laboratory of Functional Foods, Ministry of Agriculture and Rural Affairs/Guangdong Key Laboratory of Agricultural Products Processing, Guangzhou 510610, China

**Keywords:** endothelium, nobiletin, obesity, oxidative stress, vasodilation

## Abstract

**Background/Objectives**: Nobiletin, one of the main components of citrus peel, exhibits potent antioxidant, anti-inflammatory, and metabolic regulatory properties. However, its effect on obesity-associated vasculopathay remains unknown. We aim to investigate the effect of nobiletin in ameliorating oxidative stress and endothelial dysfunction induced by a high-fat diet (HFD). **Methods**: Male C57BL/6J mice were fed a HFD (60 kcal% fat) or normal chow for four months and orally administered with vehicle or nobiletin (50 mg/kg/day) for 8 weeks. Vasoreactivity in aortas was measured on a wire myograph. Primary rat aortic endothelial cells (RAECs) were isolated from Sprague-Dawley rats for in vitro study. Protein expressions were detected by Western blot. Superoxide production was determined by fluorescence imaging. **Results**: Exposure to high glucose increased the phosphorylation of JNK (Tyr185) and decreased the protein expressions of Nrf2 and HO-1, as well as downregulated the phosphorylation of AMPK and eNOS (Ser1177) in RAECs. This led to reduced nitric oxide (NO) generation and elevation of oxidative stress. High glucose induction also impaired the endothelium-dependent relaxations (EDRs) in murine aortas. These high glucose-induced impairments were restored by co-treatment of nobiletin (1 μM or 10 μM) whereas effects of nobiletin were abolished by AMPK inhibitor Compound C. The DIO-induced diabetic animal model showed increased body weight and blood pressure, imbalance of glucolipid metabolism, impaired EDRs, and elevated oxidative stress in aortas. AMPK/eNOS and Nrf2/HO-1 pathways were downregulated in aortas from DIO mice. Oral administration of nobiletin could at least partially reverse the above damage. **Conclusions**: Nobiletin ameliorates endothelial dysfunction by reducing oxidative stress and enhancing NO bioavailability upon activation of AMPK/eNOS and Nrf2/HO-1 pathways in obese diabetic mice.

## 1. Introduction

Obesity is closely associated with type 2 diabetes mellitus (T2DM), hypertension, and cardiovascular diseases (CVD) [1]. CVD contributes to over half of T2DM-related disability and death, underscoring the importance of CVD pathogenesis [2]. Endothelial dysfunction is a key early event in CVD development, characterized by impaired vasodilation, elevated oxidative stress, and reduced nitric oxide (NO) bioavailability [3]. Under physiological conditions, endothelial nitric oxide synthase (eNOS)-derived NO regulates vascular tone [4]. However, in T2DM, NO production is significantly decreased with a preserved response of vascular smooth muscle to exogenous NO donors, suggesting specific impairment in endothelial NO generation [5]. Moreover, diabetic patients often exhibit elevated triglycerides and low-density lipoprotein (LDL) cholesterol with a decreased high-density lipoprotein (HDL) cholesterol level, further exacerbating endothelial dysfunction and atherosclerosis risk [6].

Adenosine 5′-monophosphate activated protein kinase (AMPK) plays a central role in metabolic homeostasis by modulating glucose uptake, lipid metabolism, and cellular resistance to oxidative stress [7]. Notably, AMPK activation enhances eNOS phosphorylation and NO production, improving endothelial function [8]. AMPK also modulates the c-Jun *N*-terminal kinase (JNK) signaling pathway, which has been implicated in eNOS inhibition and vascular damage [9]. In parallel, the nuclear factor erythroid 2-related factor 2 (Nrf2)/hem oxygenase-1 (HO-1) pathway is a key regulator of cellular antioxidant defense, counteracting ROS-mediated vascular injury [10]. Recent studies have suggested that coordinated regulation of AMPK/eNOS and Nrf2/HO-1 signaling is critical for maintaining vascular homeostasis under metabolic stress conditions [11]. Thus, activating both AMPK and Nrf2/HO-1 pathways represents a promising strategy to mitigate diabetes-induced endothelial dysfunction [12,13].

Nobiletin (3′,4′,5,6,7,8-hexamethoxyflavone), a polymethoxylated flavone abundant in citrus peel, has been widely studied for its antioxidative, anti-inflammatory, and anticancer properties [14]. However, its vascular protective effects in the context of T2DM remain largely unexplored. Previous studies suggest that nobiletin can improve lipid metabolism and insulin resistance in obese or diabetic models [15] and associated complications through modulation of various signaling pathways, including AMPK and Nrf2 pathways [16]. Nobiletin attenuates aortic lesion development, endothelial dysfunction, and myocardial injury through inhibiting oxidative stress, ferroptosis or inflammation [16]. Previous pharmacokinetic studies have shown that nobiletin undergoes extensive metabolic biotransformation and exhibits relatively limited oral bioavailability despite its high membrane permeability, which may influence its translational applicability and dosing strategies [17].

Whether nobiletin simultaneously modulates the AMPK/eNOS and JNK/Nrf2/HO-1 pathways in vasculature has not yet been explored. Therefore, we combined vascular reactivity assays with molecular analyses and provided novel findings that nobiletin could ameliorate endothelial dysfunction through coordinated regulation of the AMPK/eNOS and JNK/Nrf2/HO-1 pathways. This study provides mechanistic evidence supporting the protective effect of nobiletin against obesity-associated vascular complications.

## 2. Materials and Methods

### 2.1. Animal Treatment

Male C57BL/6 mice were housed under controlled conditions (22 °C; 12 h light/dark cycle) with free access to food and water. All animal experiments were approved by the Animal Research Ethics Committee of University of Macau (UMARE-024-2021). Fifteen mice (18–22 g) at six weeks old were randomly divided into three groups (five mice per group) using a random number generator. The mice were fed either a normal chow diet or a high-fat diet (HFD, 60% kcal% from fat) during the 16-week experiment and being i.g. administered with 0.3% sodium carboxymethyl cellulose (CMC-Na) or nobiletin (50 mg/kg/day suspended in 0.3% CMC-Na) during the last 8 weeks. The dose of nobiletin was selected according to previous studies [16]. The groups included: Control (chow + 0.3% CMC-Na), DIO (HFD + 0.3% CMC-Na), and DIO + Nobiletin (HFD + nobiletin). Body weight, blood pressure and blood glucose were measured at the end of the experiment.

### 2.2. Blood Pressure Measurement

Systolic blood pressure (SBP) and diastolic blood pressure (DBP) among the three groups were measured using the CODA noninvasive blood pressure system (Kent Scientific Corporation, Torrington, CT, USA) in a tail cuff method. Mice were placed in restraining holders on a 32 °C warming pad for 10 min. The mean for five consecutive cycles was recorded as the data for a mouse.

### 2.3. Blood Glucose Determination

Body weight and fasting blood glucose (FBG) were monitored before and after the eight-week administration of vehicle or nobilin to confirm successful obese and diabetic model establishment. FBG was measured in mice upon 6 h-fasting. At the end of the experiment, oral glucose tolerance test (OGTT) and insulin tolerance test (ITT) were performed. For OGTT, mice were fasted for 6 h and administered glucose solution (1.2 g/kg) by oral gavage. For ITT, mice were fasted for 2 h and injected intraperitoneally with an insulin solution (0.5 U/kg). Blood glucose levels were measured for several time intervals at 0, 15, 30, 60, 90, and 120 min using a glucometer (Yuwell 580, Jiangsu Yuyue Medical Equipment & Supply Co., Ltd., Zhenjiang, China) by snipping the tail.

### 2.4. Plasma Biochemical Parameter Assays

Plasma was collected and used to measure lipid parameters, including triglyceride (TG, Stanbio Laboratory, USA), total cholesterol (TC, Stanbio Laboratory, Boerne, TX, USA), and low-density lipoprotein cholesterol (LDL-c, Nanjing Jiancheng Bioengineering Institute, Nanjing, China) following the manufacturer’s instructions. Absorbance at particular wavelengths was measured with a SpectraMax iD5 Multi-Mode Microplate Reader (Molecular Devices, San Jose, CA, USA).

### 2.5. Ex Vivo Culture of Murine Aortas

Aortas from C57BL/6J mice were cut into segments with a length of approximately 2 mm and cultured in an LG/DMEM (Gibco, Carlsbad, CA, USA) medium containing 1% penicillin streptomycin (PS) and 10% fetal bovine serum (FBS). The aortic segments were randomly divided into five groups: normal glucose group (NG, 5.55 mM glucose in medium plus 24.45 mM mannitol), high glucose group (HG, 30 mM glucose), low dose group (30 mM glucose plus 1 μM nobiletin), high dose group (30 mM glucose plus 10 μM nobiletin), and inhibitor group (30 mM glucose, 10 μM nobiletin, and 5 μM Compound C). Mannitol and glucose were dissolved in ddH_2_O. Nobiletin and Compound C were dissolved by DMSO. After 48 h incubation, the murine aortic segments were mounted on a chamber of the Multi Myograph System (Danish Myo Technology, Aarhus, Denmark) filled with Krebs buffer and 95% O_2_/5% CO_2_ at 37 °C. Isometric tension changes were measured.

### 2.6. Isometric Force Measurement by Wire Myograph

Wire myograph experiments were performed for both in vivo and ex vivo aortic segments. Arterial rings were mounted and stretched to an optimal baseline tension of 3 mN, followed by a 60 min equilibration. Vessel contractility was confirmed by 60 mM KCl. Endothelium-dependent relaxations (EDRs) were assessed by pre-contracting the rings with 3 μM phenylephrine (Phe; Sigma Aldrich, St. Louis, MO, USA), followed by gradient addition of 0.003–10 μM acetylcholine (ACh; Sigma Aldrich). Tension changes were recorded, and relaxation responses were expressed as the percentage of Phe-induced contraction. Finally, endothelium-independent relaxations were evaluated using sodium nitroprusside (SNP; Sigma Aldrich) in the presence of NG-nitro-L-arginine methyl ester (L-NAME; Sigma Aldrich), a nitric oxide synthase (NOS) inhibitor.

### 2.7. Primary Cell Culture

Aortas from Sprague-Dawley (SD) rats were isolated. The aortas were digested with 0.2% collagenase solution (Type 1A; Sigma Aldrich) at 37 °C for 15 min. The detached endothelial cells were pelleted by centrifugation and cultured with RPMI 1640 medium containing 10% FBS and 1% PS in a humidified incubator with 5% CO_2_ and 37 °C. RAECs were divided into five groups as the ex vivo experiment.

### 2.8. Cell Viability Analysis

RAECs (6 × 10^3^ cells/well) were seeded in 96-well plates and treated with DMSO or various concentrations of nobiletin (0–100 μM) for 48 h. Cell viability was assessed by a 3-(4,5-dimethylthiazol-2-yl)-2,5-diphenyltetrazolium bromide (MTT) assay. The absorbance was measured at 490 nm using a SpectraMax iD5 Multi-Mode Microplate Reader (Molecular Devices).

### 2.9. Western Blot Analysis

Protein was extracted from RAECs using a RIPA lysis buffer (Beyotime, Shanghai, China) supplemented with 1% phosphatase inhibitor cocktail (Thermo Scientific, Waltham, MA, USA) and 1% phenylmethylsulfonyl fluoride (PMSF; Thermo Scientific). On the other hand, murine aortas were lysed and homogenized in RIPA buffer containing protease inhibitor cOmplete cocktail (Sigma Aldrich) and phosphatase inhibitors PhosSTOP (Sigma Aldrich). Protein concentrations were determined using a BCA assay kit (Beyotime, China).

Proteins (15 μg) were denatured in SDS/PAGE loading buffer at 99 °C for 8 min, separated on 10% SDS-PAGE gels, and transferred to PVDF membranes (Bio-Rad, Hercules, CA, USA). Membranes were blocked for 1.5 h and then probed overnight at 4 °C with primary antibodies against GAPDH (Proteintech, Rosemont, IL, USA, cat: 60004-1-Ig), JNK (Cell Signaling Technology, Danvers, MA, USA, cat: 9252), p-JNK (Tyr185, Proteintech, cat: 80024-1-RR), AMPKα (Cell Signaling Technology, cat: 2532), p-AMPKα (Thr172, Cell Signaling Technology, cat: 2535), eNOS (Proteintech, cat: 27120-1-AP), p-eNOS (Ser1177, Cell Signaling Technology, cat: 9570), Nrf2 (Proteintech, cat: 16396-1-AP), and HO-1 (Cell Signaling Technology, cat: 43966). After washing with TBST, membranes were incubated with corresponding secondary antibodies (anti-rabbit, Beyotime, A0208; anti-mouse, Beyotime, A02616) for 1.5 h at room temperature. Protein bands were visualized by SupersignalTM West Femto Highest Sensitivity Substrate (Thermo Scientific). The bands were photographed by ChemiDocTM MP Imaging System (BIO-RAD, Hercules, CA, USA) and quantified using Image Lab 6.1 software (BIO-RAD). Band intensities were quantified using Image Lab software. Target protein expression levels were normalized to GAPDH, while phosphorylated protein levels were normalized to their corresponding total protein levels.

### 2.10. Measurement of NO Release

The cultured medium from RAECs in 6-well plates was collected. Griess Reagent I and Griess Reagent II (Beyotime) were used to assess the NO release level. Absorbance was detected at 540 nm using SpectraMax iD5 Multi-Mode microplate reader. The NO release level was normalized to the protein concentration of corresponding RAECs in the well and then compared to the control group.

### 2.11. Detection of ROS Production

Murine aortic segments were embedded in optimal cutting temperature (OCT) compound (Tissue-Tek) and sectioned at 10 μm thickness using a Leica CM1000 cryostat (Leica Microsystems, Wetzlar, Germany). Aortic ring sections and cultured RAECs were incubated in dark with 5 µM dihydroethidium (DHE; Molecular Probes, Eugene, OR, USA) for 20 min. After incubation, samples were washed three times with PBS to remove excessive dye. Fluorescence signals were captured using an Incucyte S3 Live-Cell Analysis System (Sartorius, Ann Arbor, MI, USA) and a Leica TCS SP8 Confocal Laser Scanning Microscope (Leica Microsystems). ROS fluorescence intensity was quantified using ImageJ software (National Institutes of Health, Bethesda, MD, USA, Version 1.53) and normalized to the control group.

### 2.12. Statistical Analysis

All data shown as mean ± SEM were analyzed by the GraphPad Prism 10 software, Image Lab software, ImageJ software, and LabChart Reader software (ADInstruments, Dunedin, New Zealand, Version 8). Relaxation in murine aortas was presented as % of Phe-induced contraction. The negative logarithm of ACh concentration causing 50% of the maximum response (*p*D_2_) and the maximum ACh-induced relaxation (*E*_max_%) were calculated by GraphPad Prism. Prior to statistical analysis, data normality was assessed using the Shapiro–Wilk test. Significant differences between the experimental groups were determined using two-way ANOVA. A priori defined pairwise comparisons between the control and the model groups, and between the model and treatment groups were performed using Bonferroni-adjusted tests. *p* < 0.05 was regarded as statistically significant difference. Every experiment was conducted at least three times.

## 3. Results

### 3.1. Effect of Nobiletin on Cell Viability, NO Release and Superoxide Production in RAECs

**Figure 1:**
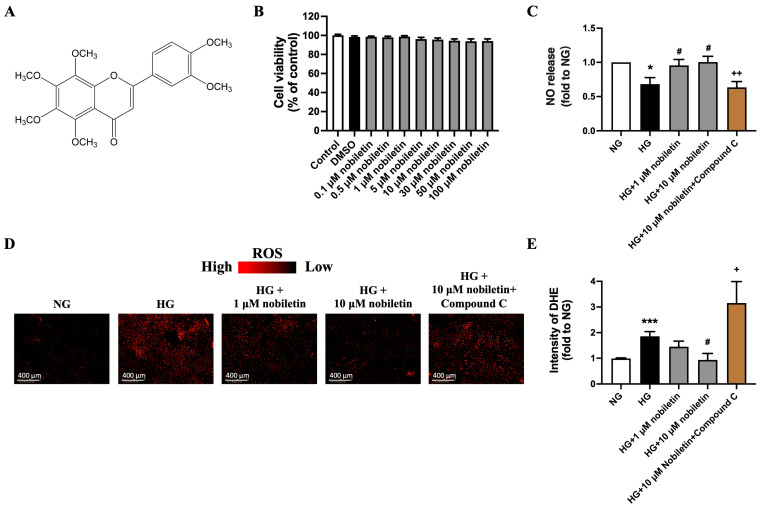
Effect of nobiletin on cell viability, NO release and superoxide generation in RAECs. (**A**) Chemical structure of nobiletin. (**B**) MTT assay, (**C**) NO release level determined by Griess Assay, and (**D**,**E**) superoxide generation assessed by DHE fluorescence staining in RAECs treated with high glucose (30 mM) or difference concentrations of nobiletin for 48 h. All data are mean ± SEM (*n* = 4). * *p* < 0.05, *** *p* < 0.001 vs. NG; # *p* < 0.05 vs. HG; + *p* < 0.05, ++ *p* < 0.01 vs. HG + 10 μM nobiletin.

Nobiletin (Figure 1A) at concentrations ranging from 0 to 100 μM for 48 h showed no significant effect on cell viability of RAECs (Figure 1B), indicating that all tested concentrations were safe under the experimental conditions. Based on this, 1 μM and 10 μM were selected as low and high doses, respectively, for subsequent in vitro experiments.

NO release is an important indicator of endothelial function. High glucose treatment (30 mM, 48 h) significantly reduced NO level in culture medium of RAECs, which was prevented by nobiletin at both 1 μM and 10 μM. Notably, co-treatment with the AMPK inhibitor Compound C abolished the effect of nobiletin on NO release (Figure 1C).

After treatment with high glucose, the DHE fluorescence intensity remarkably increased, suggesting an elevation of superoxide production (Figure 1D,E). Nobiletin significantly suppressed ROS accumulation in RAECs imposed by high glucose, and such suppression was reversed by co-treatment of Compound C.

### 3.2. Nobiletin Upregulates AMPK/eNOS and Nrf2/HO-1 Pathways in RAECs Treated with High Glucose

**Figure 2:**
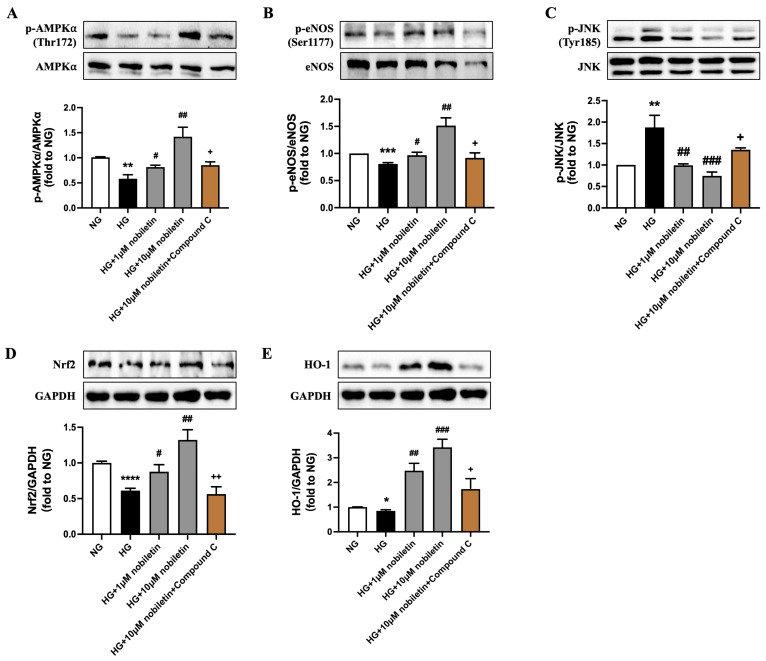
Effects of nobiletin on AMPK/eNOS and Nrf2/HO-1 signaling pathways in high glucose-induced RAECs. Representative Western blot images and quantification of the phosphorylation levels of (**A**) AMPKα at Thr172, (**B**) eNOS at Ser1177, and (**C**) JNK at Tyr185, normalized to their respective total protein levels, and expressions of (**D**) Nrf2 and (**E**) HO-1 compared to GAPDH in RAECs. All data are mean ± SEM (*n* = 4). * *p* < 0.05, ** *p* < 0.01, *** *p* < 0.001, **** *p* < 0.0001 vs. NG; # *p* < 0.05, ## *p* < 0.01, ### *p* < 0.001 vs. HG; + *p* < 0.05, ++ *p* < 0.01 vs. HG + 10 μM nobiletin.

Next, the NO signaling pathway and the master antioxidant regulators such as Nrf2 and HO-1 levels were determined. High glucose stimulation for 48 h significantly suppressed the phosphorylation of AMPKα at Thr172 (Figure 2A) and eNOS at Ser1177 (Figure 2B), as well as upregulated the phosphorylation of JNK at Tyr185 (Figure 2C). Exposure to high glucose also significantly reduced the protein levels of Nrf2 and HO-1 (Figure 2D,E). Treatment with nobiletin at both 1 μM and 10 μM markedly reversed these changes. Notably, the AMPK inhibitor Compound C abrogated the effects of nobiletin on the aforementioned protein phosphorylation and expression. The results indicate that nobiletin regulates eNOS and Nrf2/HO-1 signaling pathway possibly in an AMPK-dependent manner.

### 3.3. Nobiletin Restores Endothelial Function in High Glucose-Induced Murine Aortas Ex Vivo

**Figure 3:**
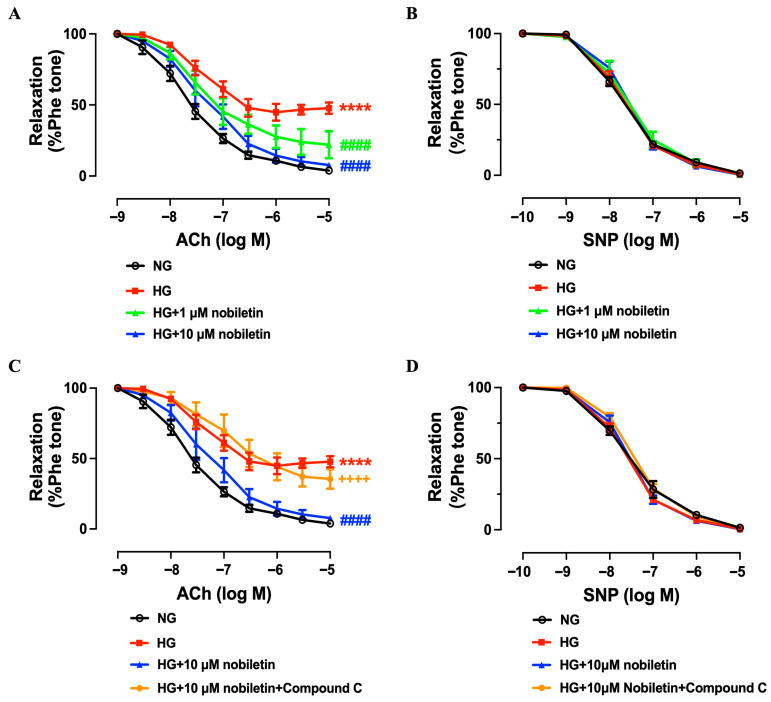
Protective effect of nobiletin on endothelium-dependent relaxations in high glucose-induced C57BL/6J murine aortas ex vivo. (**A**) Summarized data of ACh-induced endothelium-dependent relaxation of aortas. (**B**) SNP-induced endothelium-independent relaxations. (**C**) Effect of AMPK inhibitor Compound C (5 μM) on ACh-induced endothelium-dependent relaxations and (**D**) SNP-induced endothelium-independent relaxations. All data are mean ± SEM (*n* = 5). **** *p* < 0.0001 vs. NG; #### *p* < 0.0001 vs. HG; ++++ *p* < 0.0001 vs. HG + 10 μM nobiletin.

As shown in Figure 3A, ACh-induced EDRs were impaired in the HG group (*p*D_2_: 7.43; *E*_max_%: 54.20) compared to the NG group (*p*D_2_: 7.71; *E*_max_%: 94.97). Treatment with 1 μM nobiletin partially restored the impaired EDRs (*p*D_2_: 7.43; *E*_max_%: 77.37), while 10 μM nobiletin remarkably improved endothelial function (*p*D_2_: 7.38; *E*_max_%: 93.28) relative to the HG group. Nobiletin had no effects on *p*D_2_ but greatly improved *E*_max_%. In contrast, SNP-induced endothelium-independent relaxations showed no significant differences among the groups (Figure 3B). However, the addition of AMPK inhibitor compound C (5 μM) abolished the protective effect of 10 μM nobiletin on aortas exposed to high glucose (Figure 3C), proving that the effect of nobiletin on EDRs was mediated by activation of AMPK. Consistently, SNP-induced relaxations remained unaffected (Figure 3D).

### 3.4. Nobiletin Alleviates Body Weight, Blood Pressure and Glucolipid Metabolism in DIO Mice

**Figure 4:**
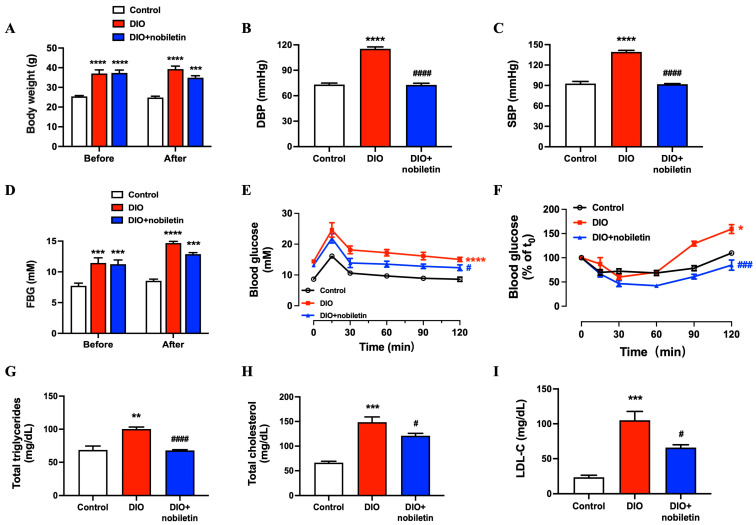
Effects of oral nobiletin administration on body weight, blood pressure and glucolipid metabolism in DIO mice. (**A**) Body weight before and after 8 weeks of nobiletin (50 mg/kg/day) administration. (**B**) Systolic blood pressure (SBP) and (**C**) diastolic blood pressure (DBP) in mice detected by tail-cuff method at the end of nobiletin treatment. (**D**) Fasting blood glucose (FBG), (**E**) oral glucose tolerance test (OGTT), and (**F**) insulin tolerance test (ITT) results after 8 weeks of nobiletin treatment in DIO mice. Plasma lipid profiles, including (**G**) total triglycerides, (**H**) total cholesterol (TC) and (**I**) low-density lipoprotein cholesterol (LDL-C). All data are mean ± SEM (*n* = 5). * *p* < 0.05, ** *p* < 0.01, *** *p* < 0.001, **** *p* < 0.0001 vs. control; # *p* < 0.05, ### *p* < 0.001, #### *p* < 0.0001 vs. DIO.

Since nobiletin had a direct effect on improving vascular function ex vivo, the vascular protective effects of nobiletin were further evaluated in vivo in DIO diabetic mice orally administered with nobiletin (50 mg/kg/day) for 8 weeks. As shown in Figure 4A, after 16-week high-fat diet (HFD), DIO mice exhibited a marked increase in body weight compared to the control group, which was mildly but not significantly reduced by nobiletin treatment. In addition, the elevated blood pressures of DIO mice were significantly reduced after nobiletin administration to levels comparable to the control group (Figure 4B,C).

The FBG level of HFD-treated mice was markedly higher than that of control mice fed with normal diet, confirming the successful establishment of diabetic model; nevertheless, eight-week treatment of nobiletin showed no significant effect on FBG level in DIO mice (Figure 4D). As shown in OGTT (Figure 4E), blood glucose level was higher in the DIO group than in the control and nobiletin-treated group at all time points. Although it remained slightly elevated in the nobiletin group compared to the control group, the blood glucose level was significantly lower than that in DIO group, implying a moderate improvement in glucose tolerance. Similarly, ITT results revealed that insulin sensitivity was impaired in the DIO group but was notably improved after nobiletin treatment (Figure 4F).

Furthermore, DIO mice suffered from hyperlipidemia manifested by the increasing levels of total triglycerides (TG, Figure 4G), total cholesterol (TC, Figure 4H), and low-density lipoprotein cholesterol (LDL-C, Figure 4I) in plasma compared with control mice. Such lipid abnormalities were partially ameliorated by oral administration of nobiletin.

### 3.5. Nobiletin Ameliorates Endothelial Dysfunction and Oxidative Stress in DIO Mice

**Figure 5:**
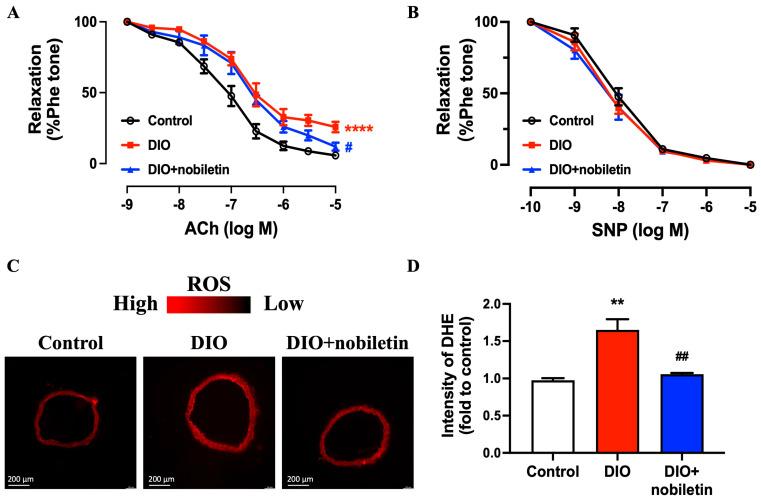
Oral administration of nobiletin alleviates endothelial function and oxidative stress in DIO mice. (**A**) ACh-induced relaxation and (**B**) SNP-induced relaxation of aortas from the three groups. (**C**) Representative images of DHE fluorescence staining of aortas and (**D**) quantification of DHE fluorescence intensity. All data are mean ± SEM (*n* = 5). ** *p* < 0.01, **** *p* < 0.0001 vs. control; # *p* < 0.05, ## *p* < 0.01 vs. DIO.

Mice on HFD for 16 weeks exhibited impaired EDRs (*p*D_2_: 6.80; *E*_max_%: 74.13) in aortas as compared with the control lean mice (*p*D_2_: 7.14; *E*_max_%: 95.57), while nobiletin treatment effectively improved the endothelial function (*p*D_2_: 6.68; *E*_max_%: 89.63) (Figure 5A). Nobiletin significantly improved *E*_max_% but not *p*D_2_. In contrast, endothelium-independent relaxations showed no difference among the three groups (Figure 5B). The images of DHE fluorescence staining manifested that ROS was significantly increased in the aortas of DIO mice and was attenuated following oral administration of nobiletin (Figure 5C,D).

### 3.6. Nobiletin Treatment Upregulates AMPK/eNOS and Nrf2/HO-1 Signaling Pathway

**Figure 6:**
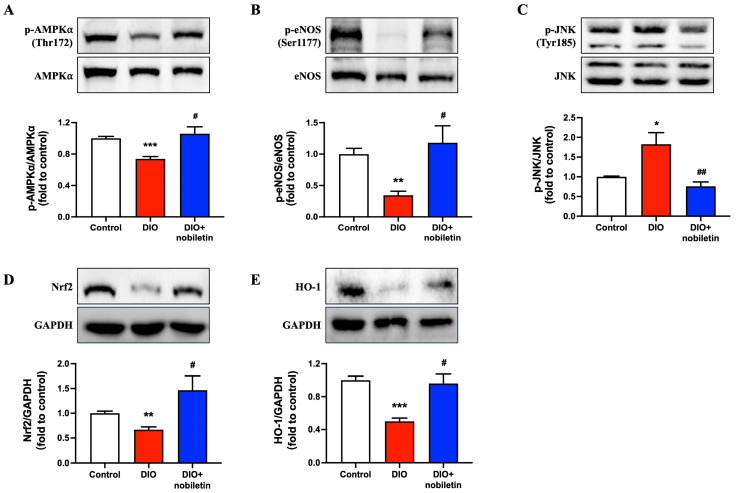
Oral administration of nobiletin activates AMPK/eNOS and Nrf2/HO-1 signaling pathways. Representative Western blots and quantification for phosphorylation of (**A**) AMPKα (Thr172), (**B**) eNOS (Ser1177) and (**C**) JNK (Tyr185) with their corresponding total protein in murine aortas. Representative Western blots and quantification of (**D**) Nrf2 and (**E**) HO-1 expression compared to GAPDH in aortas. All data are mean ± SEM (*n* = 5). * *p* < 0.05, ** *p* < 0.01, *** *p* < 0.001 vs. control; # *p* < 0.05, ## *p* < 0.01 vs. DIO.

The phosphorylation level of AMPKα at Thr172 and eNOS at Ser1177 was significantly downregulated in aortas of DIO mice and was reversed by nobiletin administration (Figure 6A,B). The phosphorylation of JNK at Tyr185 was enhanced (Figure 6C) whilst expressions of Nrf2 and HO-1 were diminished in the aortas of DIO mice (Figure 6D,E). Eight-week oral administration of nobiletin effectively restored these expressions, consistent with the results of in vitro experiments.

## 4. Discussion

Previous studies have reported that nobiletin possesses anti-diabetic effects [18]. The present study demonstrated that nobiletin not only restored glucose tolerance and insulin sensitivity, but also ameliorated blood pressure, lipid profile and vascular endothelial dysfunction associated with high-fat diet-induced obese diabetic mice. Moreover, the present results confirmed that nobiletin enhanced endothelium-derived NO production by inducing the phosphorylation of eNOS at Ser1177 dependent on AMPK activation. AMPK is the upstream modulator of multiple targets and has been recognized as a viable therapeutic target. AMPK activation by metformin enhances eNOS phosphorylation and improves endothelial function in diabetic models [19]. Baicalin, another flavonoid, also activates AMPK and restores NO bioavailability [20]. These findings imply that the activation of AMPK/eNOS signaling is beneficial for vascular function in diabetes.

In addition to regulating eNOS phosphorylation, nobiletin inhibited the activation of JNK under hyperglycemic stress. Inhibiting the JNK pathway has been shown to improve endothelial function by a variety of drugs, as for example, nebivolol and ginsenoside Rc can alleviate endothelial injury in diabetes by downregulating JNK activity [21,22]. The results of this research coincide with previous studies and support JNK as an important target for vascular endothelial injury in diabetes [23]. AMPK phosphorylation can induce the inhibition of JNK and activation of eNOS, promoting NO generation and endothelial function [9]. In addition, JNK activation is indirectly involved in the downregulation of eNOS phosphorylation, leading to reduced NO production and endothelial dysfunction [24]. The modulation of AMPK and JNK by nobiletin highlights its dual regulatory role in promoting endothelial homeostasis.

Oxidative stress is another critical factor contributing to endothelial injury in diabetes. Nrf2 is a key modulator for oxidative stress, inducing antioxidant genes and proteins including HO-1. Nrf2 is constitutively expressed at low levels under basal conditions and is tightly regulated by Kelch-like ECH-associated protein 1 (KEAP1)-mediated degradation, while HO-1 exhibits low basal expression and is strongly inducible under stress conditions [25]. The Nrf2/HO-1 pathway has been shown to be activated by multiple phytochemicals with vascular benefits [26]. For instance, broccoli-derived sulforaphane activates Nrf2 to suppress oxidative stress and diabetes-induced complications [27]. Resveratrol also increases Nrf2 nuclear translocation and enhances antioxidant capacity in endothelial cells [28]. Our study confirmed that high glucose increased ROS production in endothelial cells, while nobiletin significantly reduced intracellular ROS levels. This antioxidant effect was accompanied by the upregulation of Nrf2 and HO-1 expression, which are key regulators of the cellular antioxidant defense system. Moreover, compound C blocked these effects, indicating that the antioxidant action of nobiletin is also AMPK-dependent. It is also reported that oxidative stress results in the activation of JNK and inhibition of JNK upregulates Nrf2/HO-1 signaling to exert an antioxidant effect [29]. Taken together, these findings highlight the ability of nobiletin to activate AMPK and thereby induce eNOS/NO and Nrf2/HO-1 antioxidant signaling pathways. The increased NO bioavailability and suppressed oxidative stress contribute to the protective effects of nobiletin in diabetes. However, some studies suggest that persistent overactivation of Nrf2 may impair metabolic flexibility, underscoring the importance of regulated, rather than constitutive Nrf2 activation [30].

Endothelium-dependent relaxations (EDRs) in murine aortas are mainly mediated by NO and rely on the functional integrity of endothelial cells. The vascular endothelial protective effects of nobiletin were further validated in ex vivo and in vivo wire myographs. Our results showed that nobiletin restored EDRs of aortas exposed to high glucose, and this effect was reversed by compound C, supporting the involvement of AMPK. In DIO-induced diabetic mice, nobiletin not only improved glucose tolerance and insulin sensitivity, but also improved lipid profiles and lowered blood pressure. These systemic effects might contribute to the enhanced vascular endothelial function. However, a potential interplay between metabolic regulation and endothelial protection involved in nobiletin bioactivities has to be validated by further investigation. Notably, the ambient glucose and lipid levels in culture medium were kept constant for model and treatment groups. The ex vivo and in vitro results strongly supported that nobiletin exhibited direct endothelial protective effects, independent of effects on glucose or lipid metabolism observed in vivo. Some compounds show endothelial improvement without affecting metabolism. For instance, carvedilol enhances EDRs through endothelial-mediated NO release, independent of insulin resistance [31,32]. In contrast, nobiletin appeared to exert both metabolic and vascular benefits in diabetes.

Several limitations exist for the current study and remain to be investigated in future research. The upstream mechanisms responsible for AMPK activation by nobiletin remain unclear. The potential targets of nobiletin could be further explored using molecular docking to predict, and then molecular assays to validate. Pharmacokinetic, toxicological, and drug-interaction profiles also require further characterization to assess the clinical applicability and potential for combination therapies. In addition, the translational relevance of the current dosing regimen to potential human therapeutic applications remains unclear, and future studies should further evaluate human-equivalent dosing strategies for nobiletin. Notably, nobiletin exhibits limited oral bioavailability due to its poor water solubility and extensive first-pass metabolism, which should be considered when interpreting its translational potential. Although Compound C was used to investigate the involvement of AMPK signaling, further genetic approaches such as AMPK knockdown or knockout models are needed to more definitively validate AMPK dependency and exclude potential off-target effects of pharmacological inhibition. Moreover, oxidative stress in the present study was mainly evaluated by DHE fluorescence staining, which is semi-quantitative and may be influenced by experimental conditions. Additional oxidative stress-related assays, such as antioxidant enzyme activity and lipid peroxidation measurements, would provide more comprehensive evidence for the antioxidative effects of nobiletin. Since the number of animals per group was relatively limited, further experiments with larger cohorts would improve statistical power and reproducibility. Moreover, as only a single dose and short-term administration were employed in this study, future investigations should explore the effects and safety/toxicology of prolonged treatment and different dosing strategies. In addition, a normal chow-fed group treated with nobiletin was not included in the present study. Therefore, the potential effects of nobiletin under normal physiological conditions require further investigation.

In summary, our findings provide supportive evidence that nobiletin mitigates diabetes-associated endothelial dysfunction through modulation of metabolic and antioxidant mechanisms, primarily mediated by AMPK activation. By enhancing NO availability and attenuating oxidative stress, nobiletin targets critical pathological features of diabetic vascular disease. These mechanistic insights expand the current understanding of nobiletin’s pharmacological properties and support its further development as a potential healthcare product to reduce vascular complications associated with metabolic disorders.

## 5. Conclusions

In this study, the endothelial protective effect, antioxidant activity and the underlying mechanisms of nobiletin were investigated in vitro and in vivo. It was indicated that nobiletin exhibited endothelial protective effects to improve vasodilations and NO release accomplished with suppressed oxidative stress in high glucose-induced RAECs and murine aortas as well as in aortas from DIO mice. Oral administration of nobiletin also diminished high blood pressure, improved glucose tolerance and insulin sensitivity, and alleviated hyperlipidemia. The beneficial effects of nobiletin on the amelioration of endothelial dysfunction and oxidative stress were possibly related to the activation of AMPK/JNK/eNOS and Nrf2/HO-1 signaling pathways. Collectively, these findings provided experimental evidence supporting the protective effects of nobiletin against obesity-associated endothelial dysfunction in cellular and DIO mouse models. Due to the safety and efficacy of nobiletin, it might serve as a potential candidate for further investigation in the prevention or amelioration of obesity-associated endothelial dysfunction.

## Data Availability

The original contributions presented in this study are included in the article. Further inquiries can be directed at the corresponding authors.

## Abbreviations

The following abbreviations are used in this manuscript:

T2DM	Type 2 diabetes mellitus
CVD	Cardiovascular diseases
NO	Nitric oxide
eNOS	Endothelial nitric oxide synthase
AMPK	Adenosine 5′-monophosphate activated protein kinase
DIO	Diet-induced obese
HFD	High-fat diet
SBP	Systolic blood pressure
DBP	Diastolic blood pressure
FBG	Fasting blood glucose
OGTT	Oral glucose tolerance test
ITT	Insulin tolerance test
EDRs	Endothelium-dependent relaxations
Phe	Phenylephrine
ACh	Acetylcholine
SNP	Sodium nitroprusside
L-NAME	NG-nitro-L-arginine methyl ester

## References

[1] Henning R.J. (2018). Type-2 diabetes mellitus and cardiovascular disease. Future Cardiol..

[2] Safiri S., Karamzad N., Kaufman J.S., Bell A.W., Nejadghaderi S.A., Sullman M.J.M., Moradi-Lakeh M., Collins G., Kolahi A.-A. (2022). Prevalence, Deaths and Disability-Adjusted-Life-Years (DALYs) Due to Type 2 Diabetes and Its Attributable Risk Factors in 204 Countries and Territories, 1990–2019: Results From the Global Burden of Disease Study 2019. Front. Endocrinol..

[3] Wang X., He B. (2024). Endothelial dysfunction: Molecular mechanisms and clinical implications. MedComm.

[4] Tran N., Garcia T., Aniqa M., Ali S., Ally A., Nauli S.M. (2022). Endothelial Nitric Oxide Synthase (eNOS) and the Cardiovascular System: In Physiology and in Disease States. Am. J. Biomed. Sci. Res..

[5] Toda N., Imamura T., Okamura T. (2010). Alteration of nitric oxide-mediated blood flow regulation in diabetes mellitus. Pharmacol. Ther..

[6] Bardini G., Rotella C.M., Giannini S. (2012). Dyslipidemia and diabetes: Reciprocal impact of impaired lipid metabolism and Beta-cell dysfunction on micro- and macrovascular complications. Rev. Diabet. Stud..

[7] Jeon S.-M. (2016). Regulation and function of AMPK in physiology and diseases. Exp. Mol. Med..

[8] Morrow V.A., Foufelle F., Connell J.M., Petrie J.R., Gould G.W., Salt I.P. (2003). Direct activation of AMP-activated protein kinase stimulates nitric-oxide synthesis in human aortic endothelial cells. J. Biol. Chem..

[9] Rodríguez C., Muñoz M., Contreras C., Prieto D. (2021). AMPK, metabolism, and vascular function. FEBS J..

[10] Ngo V., Duennwald M.L. (2022). Nrf2 and Oxidative Stress: A General Overview of Mechanisms and Implications in Human Disease. Antioxidants.

[11] Miao L., Cheong M.S., Zhou C., Farag M., Cheang W.S., Xiao J. (2023). Apigenin alleviates diabetic endothelial dysfunction through activating AMPK/PI3K/Akt/eNOS and Nrf2/HO-1 signaling pathways. Food Front..

[12] Al-Amarat W., Abukhalil M.H., Alruhaimi R.S., Alqhtani H.A., Aldawood N., Alfwuaires M.A., Althunibat O.Y., Aladaileh S.H., Algefare A.I., Alanezi A.A. (2022). Upregulation of Nrf2/HO-1 Signaling and Attenuation of Oxidative Stress, Inflammation, and Cell Death Mediate the Protective Effect of Apigenin against Cyclophosphamide Hepatotoxicity. Metabolites.

[13] Ebrahimi R., Mohammadpour A., Medoro A., Davinelli S., Saso L., Miroliaei M. (2025). Exploring the links between polyphenols, Nrf2, and diabetes: A review. Biomed. Pharmacother..

[14] Qin Y., Yang J., Li H., Li J. (2024). Recent advances in the therapeutic potential of nobiletin against respiratory diseases. Phytomedicine.

[15] Lee Y.-S., Cha B.-Y., Choi S.-S., Choi B.-K., Yonezawa T., Teruya T., Nagai K., Woo J.-T. (2013). Nobiletin improves obesity and insulin resistance in high-fat diet-induced obese mice. J. Nutr. Biochem..

[16] Zhao C., Lai W., Li Y., Hong K., Xu Y. (2025). Potential and Mechanism of Nobiletin in Diabetes Mellitus and Associated Complications. Pharmaceuticals.

[17] Li S., Pan M.-H., Lo C.-Y., Tan D., Wang Y., Shahidi F., Ho C.-T. (2009). Chemistry and health effects of polymethoxyflavones and hydroxylated polymethoxyflavones. J. Funct. Foods.

[18] Gandhi G.R., Vasconcelos A.B.S., Wu D.T., Li H.B., Antony P.J., Li H., Geng F., Gurgel R.Q., Narain N., Gan R.Y. (2020). Citrus Flavonoids as Promising Phytochemicals Targeting Diabetes and Related Complications: A Systematic Review of In Vitro and In Vivo Studies. Nutrients.

[19] An H., Wei R., Ke J., Yang J., Liu Y., Wang X., Wang G., Hong T. (2016). Metformin attenuates fluctuating glucose-induced endothelial dysfunction through enhancing GTPCH1-mediated eNOS recoupling and inhibiting NADPH oxidase. J. Diabetes Its Complicat..

[20] Miao L., Yang Y., Dai J., Bai M., Wang Y., Cui H., Lin L., Alharbi M., Cheang W.S. (2024). Baicalin attenuates vascular inflammation and endothelial dysfunction in diabetes. Food Front..

[21] Li X., Li Q., Wu L., Wang Y. (2023). Nebivolol Alleviates Vascular Endothelial Insulin Resistance by Inhibiting Endoplasmic Reticulum Stress. Int. Heart J..

[22] Wang Y., Fu W., Xue Y., Lu Z., Li Y., Yu P., Yu X., Xu H., Sui D. (2021). Ginsenoside Rc Ameliorates Endothelial Insulin Resistance via Upregulation of Angiotensin-Converting Enzyme 2. Front. Pharmacol..

[23] Bretón-Romero R., Feng B., Holbrook M., Farb M.G., Fetterman J.L., Linder E.A., Berk B.D., Masaki N., Weisbrod R.M., Inagaki E. (2016). Endothelial Dysfunction in Human Diabetes Is Mediated by Wnt5a-JNK Signaling. Arterioscler. Thromb. Vasc. Biol..

[24] Park J.H., Park M., Byun C.J., Jo I. (2012). c-Jun N-terminal kinase 2 phosphorylates endothelial nitric oxide synthase at serine 116 and regulates nitric oxide production. Biochem. Biophys. Res. Commun..

[25] Loboda A., Damulewicz M., Pyza E., Jozkowicz A., Dulak J. (2016). Role of Nrf2/HO-1 system in development, oxidative stress response and diseases: An evolutionarily conserved mechanism. Cell. Mol. Life Sci..

[26] He W.J., Lv C.H., Chen Z., Shi M., Zeng C.X., Hou D.X., Qin S. (2023). The Regulatory Effect of Phytochemicals on Chronic Diseases by Targeting Nrf2-ARE Signaling Pathway. Antioxidants.

[27] Bai Y., Wang X., Zhao S., Ma C., Cui J., Zheng Y. (2015). Sulforaphane Protects against Cardiovascular Disease via Nrf2 Activation. Oxid. Med. Cell. Longev..

[28] Ungvari Z., Bagi Z., Feher A., Recchia F.A., Sonntag W.E., Pearson K., de Cabo R., Csiszar A. (2010). Resveratrol confers endothelial protection via activation of the antioxidant transcription factor Nrf2. Am. J. Physiol. Heart Circ. Physiol..

[29] Khan M.S., Khan A., Ahmad S., Ahmad R., Rehman I.U.R., Ikram M., Kim M.O. (2020). Inhibition of JNK Alleviates Chronic Hypoperfusion-Related Ischemia Induces Oxidative Stress and Brain Degeneration via Nrf2/HO-1 and NF-κB Signaling. Oxid. Med. Cell. Longev..

[30] Tebay L.E., Robertson H., Durant S.T., Vitale S.R., Penning T.M., Dinkova-Kostova A.T., Hayes J.D. (2015). Mechanisms of activation of the transcription factor Nrf2 by redox stressors, nutrient cues, and energy status and the pathways through which it attenuates degenerative disease. Free Radic. Biol. Med..

[31] Nishioka K., Nakagawa K., Umemura T., Jitsuiki D., Ueda K., Goto C., Chayama K., Yoshizumi M., Higashi Y. (2007). Carvedilol improves endothelium-dependent vasodilation in patients with dilated cardiomyopathy. Heart.

[32] Refsgaard J., Thomsen C., Andreasen F., Gøtzsche O. (2002). Carvedilol does not alter the insulin sensitivity in patients with congestive heart failure. Eur. J. Heart Fail..

